# COMBINA: The EAAD four-level approach for depression and suicide prevention and wellbeing promotion in the community and vulnerable populations. A cross-country study protocol from the MENTBEST project

**DOI:** 10.1371/journal.pone.0352598

**Published:** 2026-07-02

**Authors:** Bridget Hogg, Evelien Coppens, Daniela L. Gatto, Cristina de Córdoba-Gil, José Luis Ayuso Mateos, Keti Bakiu, Aileen Callanan, Arlinda Cerga Pashoja, Virgínia Conceição, Evgenia Dandi, Maria Faurholt-Jepsen, Christina Katsoridou, Almas Khan, Jeroen Luyten, Kerry R. McGreevy, Rainer Mere, Darragh O’Shea, Georgia Papaioannou, Gentiana Qirjako, Hanna Reich, Peeter Värnik, Airi Värnik, Ana B. Vivas, Ella Arensman, Chantal Van Audenhove, Ricardo Gusmão, Benedikt L. Amann, Katharina Schnitzspahn, Ulrich Hegerl

**Affiliations:** 1 Hospital del Mar Research Institute, Barcelona, Spain; 2 Centro de Investigación Biomédica en Red de Salud Mental, Instituto de Carlos III, Madrid, Spain; 3 Centre for Care Research and Consultancy, KU Leuven, Leuven, Belgium; 4 Department of Medicine and Life Sciences (MELIS), Phd Programme in Biomedicine Universitat Pompeu Fabra, Barcelona, Spain; 5 Department of Psychiatry, Universidad Autónoma de Madrid (UAM), Madrid, Spain; 6 Instituto de Investigación Sanitaria del Hospital Universitario La Princesa, Madrid, Spain; 7 Community Centre for Health and Wellbeing, Tirana, Albania; 8 National Suicide Research Foundation, University College Cork, Cork, Ireland; 9 St Marys University, Twickenham, United Kingdom; 10 EPIUnit - Institute of Public Health, University of Porto, Portugal; 11 Laboratory for Integrative and Translational Research in Population Health (ITR), University of Porto, Porto, Portugal; 12 South East European Research Center (SEERC); Department of Psychology, University of York Europe Campus, CITY ULE, Thessaloniki, Greece; 13 Copenhagen Affective Disorder Research Center (CADIC), Psychiatric Center Copenhagen, Frederiksberg, Denmark; 14 Department of Clinical Medicine, Faculty of Health and Medical Sciences, University of Copenhagen, Denmark; 15 School of Public Health, University College Cork, Cork, Ireland; 16 Leuven Unit for HTA Research, Leuven Institute for Healthcare Policy, & Department of Public Health and Primary Care, KU Leuven, Leuven, Belgium; 17 Department of Public Health & Primary Care, LUCAS, Leuven, Belgium; 18 Tallinn University, School of Governance, Law and Society (SOGOLAS), Tallinn, Estonia; 19 Estonian-Swedish Mental Health and Suicidology Institute (ERSI), Tallinn, Estonia; 20 Hull York Medical School, University of York, United Kingdom; 21 University of Medicine, Tirana, Albania; 22 Research Centre of the German Foundation for Depression and Suicide Prevention, Leipzig, Germany; 23 Department for Psychiatry, Psychosomatics and Psychotherapy, University Hospital, Goethe University Frankfurt, Frankfurt am Main, Germany; 24 Institute of Mental Health, Hospital del Mar Barcelona, Barcelona, Spain; 25 Department of Medicine and Life Sciences (MELIS), Universitat Pompeu Fabra, Barcelona, Spain; 26 European Alliance Against Depression, Leipzig, Germany; PLOS: Public Library of Science, UNITED KINGDOM OF GREAT BRITAIN AND NORTHERN IRELAND

## Abstract

Depression and suicide are leading public health problems requiring complex multilevel interventions. This study protocol details the COMBINA trial, which expands the evidence-based European Alliance Against Depression (EAAD) Community-Based 4-level intervention to also focus on improving wellbeing and tailored to five groups with increased vulnerability to depression: young people, older people, migrants/refugees, the long-term unemployed, and people with an existing mental health condition. In this prospective, non-randomised controlled trial, the COMBINA project will be newly implemented during a 24-month period, from late 2024 to late 2026, in five regions in Albania, Estonia, Greece, Ireland, and Spain. These regions will be compared to five control regions in the same countries, chosen to reflect a similar context, size, and sociodemographic characteristics. Main outcomes are a comparison between the intervention and control regions in the rates of deaths by suicide and hospitalisations for suicide attempts and the levels of wellbeing, depressive symptoms, anxiety symptoms, depression-related stigma, and willingness to seek psychological help in the general population. A process evaluation and economic evaluation will also be conducted. This intervention protocol also details the steps taken to promote successful implementation across a range of different cultural and regional contexts, with challenges including different existing mental health resources and differing capacities for the population to use digital tools. The COMBINA project includes co-creation, whereby materials and implementation strategy were designed with people from the vulnerable groups. The trial is funded by the European Union (grant agreement no. 101080651 and carried out according to the Declaration of Helsinki. Ethical approval has been obtained in all participating countries. The results will be published in peer-reviewed academic journals, presented at scientific meetings and disseminated through COMBINA stakeholders, with participation from the co-creators. This trial protocol (version 1.1, 24/10/2024) was registered in the ISRCTN registry (ISRCTN10521127) on 14/11/2025 (https://www.isrctn.com/ISRCTN10521127).

## Introduction

Depression remains a leading cause of disability worldwide, and its burden continues to rise in high-income regions. Major depressive disorder affects approximately 6.5-6.9% of the population each year [1,2] and poses substantial challenges for health systems [3,4], leading to a high clinical as well as economic burden of disease [5]. Furthermore, depressive disorders account for approximately 50%–60% of suicide deaths globally, making depression the most common underlying mental disorder associated with suicide [6–8]. Each year, an estimated 1,000,000 suicides occur worldwide [9], while attempted suicides are estimated to be about 20 times higher [10].

Suicide prevention is a public health issue driven by social determinants and individual and other risk factors [11], meaning it needs to be tackled at a community level, requiring action at different levels across the community [12,13]. A recent systematic review, which synthesised 47 studies published between 2010 and 2020, identified the European Alliance Against Depression (EAAD)’s community-based 4-level intervention as the most promising community-based intervention in terms of preventing suicide [14]. The EAAD intervention includes activities at four levels: 1) Primary health care, 2) General population, 3) Community facilitators, and 4) Patients and their relatives [15], and has been considered as a best practice intervention by the World Health Organisation and European Commission [10,16]. Since 2008, the concept has been implemented in more than 120 regions in 15 countries within Europe and beyond [17,18].

Despite growing evidence for the effectiveness of multi-level community interventions such as the EAAD model, their systematic implementation and long-term sustainability remain limited across Europe. Challenges to effective translation of evidence-based mental health interventions include insufficient adaptation to local contexts [19,20], limited engagement of vulnerable populations [21,22], and fragmentation between health and community sectors [23–26].

Within this context, the COMBINA study builds on the EAAD’s evidence-based 4-level approach by tailoring it to vulnerable groups and providing flexibility for implementation across different contexts. The 4-level intervention is adapted to meet the specific needs of five vulnerable groups who are often underserved by conventional mental health services: young people, older people, long-term unemployed, migrants and refugees, and people with current or past mental disorders. These vulnerable groups were chosen due to the risk of being underserved by existing mental health services. In terms of young people, the peak age of onset for mental disorders is 14.5 years old [27], yet the majority of young people do not seek professional help for mental health problems [28], and only approximately one third of adolescents with mental disorders receive treatment [29]. Conversely, older people have different specific needs, such as family-centred care [30], and are at risk of depression due to unmet healthcare needs [31]. Meanwhile, being unemployed is associated with higher rates of depression [32], and is also a risk factor for worse outcomes in depression [33] and for suicide [34]. Similarly, refugees and displaced people are at greater risk of a range of mental health conditions [35–37]. In voluntary migrants, a review showed consistent evidence for deterioration of mental health over time [38] and a lower sense of belonging in migrants is linked to increased anxiety and depression [39]. Finally, those with depression are at greater risk during societal change [40] and are likely to be undertreated [41], with a recent review finding only between 9% and 35% of people in Europe receive even minimal treatment for depression, depending on the country [42].

The intervention will be trialled across five regions within Albania, Estonia, Greece, Ireland, and Spain. These regions have diverse contexts and mental healthcare systems, requiring adaptation not only to vulnerable groups but to a range of new environments. The COMBINA project is also innovative in broadening the focus of the EAAD community-based 4-level intervention to improve wellbeing by including a focus on subclinical symptoms of anxiety and depression. Improved wellbeing has been shown to be a protective factor for depression [43,44], and psychological interventions for depressive symptoms not meeting clinical significance can improve them and prevent an episode of a depressive disorder [45,46]

The COMBINA trial, which forms part of the “MENTBEST: Protecting mental health in times of change” study funded by the European Union, will apply EAAD’s synergistic, multilevel, multicomponent community intervention, using a controlled non-randomized prospective design. The intervention will be implemented in five European regions. Each region will be paired with a comparable control region where no intervention activities will take place. The overall aims are to promote wellbeing and reduce depressive symptoms, suicide attempts, and the number of deaths by suicide, in the general population and among the five identified vulnerable groups. Furthermore, the intervention aims to reduce depression-related stigma and improve attitudes towards help-seeking for mental disorders.

## Methods

Although the study is not a randomised controlled trial, the evaluation design described in this manuscript is written in line with the StaRI reporting guidelines [47] and the SPIRIT checklist for trial protocols has been filled in, with items exclusively for randomised controlled trials marked as not applicable, and can be seen in Supporting Information S1 File.

### Study aims and design

**Fig 1:**
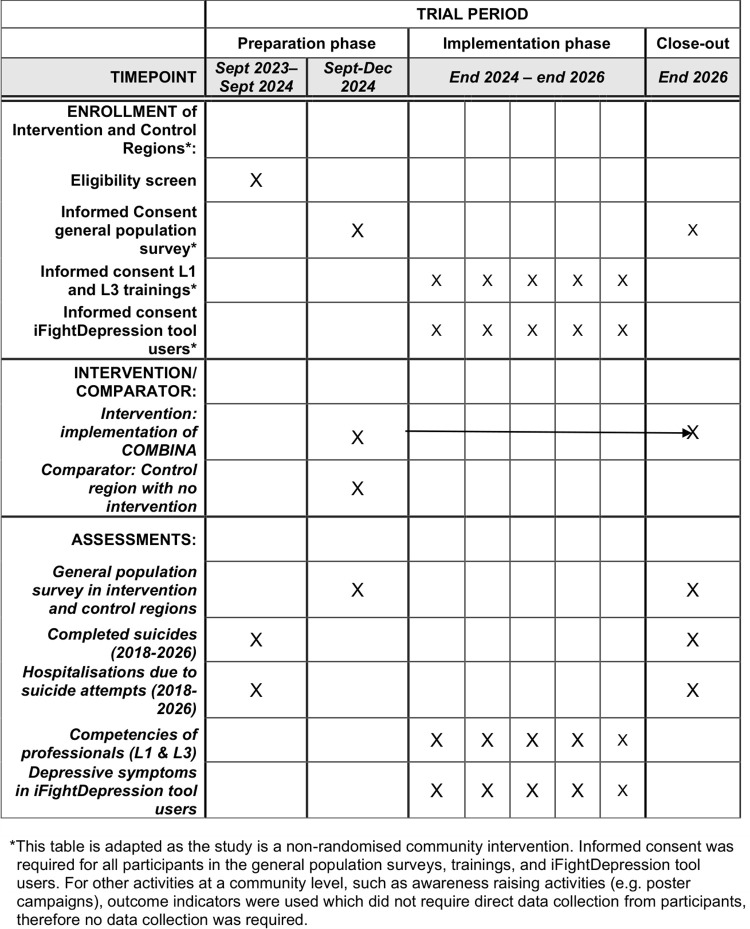
SPIRIT Participant timeline: Schedule of enrolment, interventions, and assessments.

**Fig 2:**
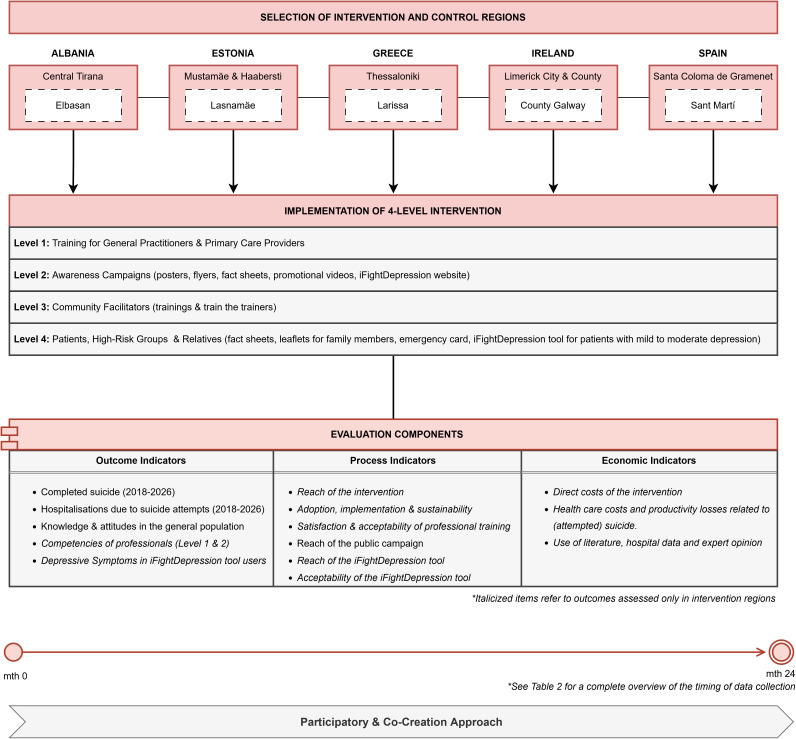
Protocol Overview. Cross-country study protocol flowchart illustrating the main steps: region selection (control regions are shown within boxes with dashed borders), implementation of the 4-level intervention, evaluation components, and timeline (0–24 months; Mth = months).

A SPIRIT schedule of enrolment, interventions, and assessment can be seen in Fig 1, adapted to the multi-level non-randomised nature of our study. More detail regarding the overview of the study can also be seen in Fig 2. The study has three primary aims:

1. Process evaluation: to comprehensively investigate the context-related implementation process of the intervention, including fidelity, reach, adoption, acceptability, and contextual facilitators and barriers across diverse European settings (Albania, Estonia, Greece, Ireland, and Spain).
2. Outcome evaluation: To examine whether the expanded four-level intervention achieves its intended outcomes through a controlled, cross-country design. The evaluation will focus on clinical outcomes (e.g., reductions in suicidal behaviour and depressive symptoms) and non-clinical outcomes (e.g., improvements in wellbeing, self-reported mental health, and attitudes toward depression and help-seeking). Additionally, pre-post assessments without a control group will be included as well.
3. Economic evaluation: to determine the cost-effectiveness of the intervention, expressed as the observed incremental cost per unit of outcome gained: cost per death by suicide avoided, per suicide attempt avoided and per percentage point improvement in selected clinical and subclinical outcomes derived from the population surveys conducted in each site.

The effectiveness of the expanded community-based 4-level intervention will be evaluated through a prospective controlled cross-country design across five European countries – Albania, Estonia, Greece, Ireland, and Spain. In each country, one intervention region and one comparable control region will be selected to allow for between-group comparisons. Primary clinical outcomes will be collected in both intervention and control regions to assess changes in depressive symptoms, suicidal behaviour, and wellbeing. Additionally, intermediate clinical and service-related outcomes will be collected in the intervention regions only to explore mechanisms of change and evaluate the broader system-level impact of the intervention.

The process evaluation will be conducted in both the intervention and control regions, using a mixed-methods approach that integrates both quantitative and qualitative data collection measures.

### Study regions and populations

During the study, data will be collected from multiple regions and populations targeted by the expanded community-based 4-level intervention. This protocol (version 1.1, dated 24.10.2024) has been registered in the ISRCTN registry under number ISRCTN10521127, registered 14/11/2025¸ https://www.isrctn.com/ISRCTN10521127).

### Regions

Each participating country selected a region which was feasible and had a suitable context for implementing a large-scale community intervention. A control region was chosen to match as closely as possible the characteristics of the chosen intervention region in terms of size, level of urbanisation, and sociodemographic characteristics, to minimise selection bias. Baseline sociodemographic and mental health indicators will be collected for both regions to assess comparability and to adjust for pre-existing differences in the analyses. Population sizes were matched as closely as feasible, ranging from approximately 117,538–350,000 inhabitants across the five participating countries (see Table 1).

**Table 1:** Overview of intervention and control sites.

Country	Intervention area (Number of inhabitants)	Control area (Number of inhabitants)
Albania	Central Tirana (350,000)	Elbasan (252,719)
Estonia	Mustamäe & Haabersti (117,538)	Lasnamäe (119,092)
Greece	Thessaloniki municipality (324,766)	Larissa (144,651)
Ireland	Limerick City & County Council (209,536)	County Galway (277,737)
Spain	Santa Coloma de Gramenet (119,195)	Sant Marti (242,752)

### Populations

The 4-level intervention is aimed at four main populations: Level 1 – General Practitioners and other primary healthcare providers; Level 2 – the general population, including the five vulnerable groups; Level 3 – community gatekeepers who work with the general population and the five vulnerable groups (e.g., police officers, social workers, teachers, youth workers, carers in geriatric settings, religious leaders, pharmacists); and Level 4 – people with depression and their families.

### Vulnerable groups

Within the scope of this project, the vulnerable groups are defined as per the following definitions: 1) **Young people** are defined as those between the ages of 15 and 24 years [48]; 2) **Older people** are defined as 65 + years [49]; 3) **Long-term unemployed** are defined as people who have been unemployed for twelve months or more [50]; 4) **Migrants/refugees**: a migrant is defined as someone who changes his or her country of usual residence, irrespective of the reason for migration or legal status [51], while a refugee is defined as someone who is unable or unwilling to return to their country of origin owing to a well-founded fear of being persecuted for reasons of race, religion, nationality, membership of a particular social group, or political opinion [52]; 5) **People with an existing mental disorder** include all people who have a current or previous diagnosis of depression or any other mental disorder, according to the Diagnostic and Statistical Manual of Mental Disorders or International Classification of Diseases [53,54], prior to the beginning of the trial. However, the definitions of the vulnerable groups provide a framework for the development of the project but are to be applied in a flexible and pragmatic way, and as there is a concurrent focus on the general population, no one is excluded from the project for not strictly meeting one of the aforementioned definitions.

In order to ensure that the adaptations to the vulnerable groups are trialled thoroughly in the intervention, each vulnerable group is a specific focus for at least three of the five intervention countries, as can be seen in Table 2.

**Table 2:** Vulnerable groups as a focus for the intervention by country.

	People with lived experience of mental disorder	Young people	Elderly	Unemployed	Migrants/Refugees
**Albania**	X	X	X		
**Estonia**	X	X		X	X
**Greece**	X	X	X		X
**Ireland**	X	X	X	X	
**Spain**	X			X	X

### Intervention and materials

The COMBINA project combines the EAAD community-based 4-level intervention for preventing suicide and reducing depression with innovative resources for improving wellbeing, and tailors the intervention towards the general population and the five identified vulnerable groups.

### Co-creation

A distinguishing feature of COMBINA is its participatory and co-creation approach, ensuring that the public and those affected by depression are involved in the design, or conduct, or reporting, or dissemination plans of this research. Co-creation ensures that intervention components are culturally and contextually relevant, fostering ownership and sustainability within each implementation site. Through iterative engagement with co-creators who represent the identified vulnerable groups and local stakeholders, the intervention is adapted to reflect the priorities and realities of local communities. Evidence increasingly supports co-creation as a mechanism that enhances feasibility, acceptability, contextual fit, and sustainability of health and mental-health interventions [22,55,56]. Within COMBINA, co-creation is embedded across all stages, from design to delivery and evaluation, representing both a methodological innovation and an implementation strategy.

### Materials

**Fig 3:**
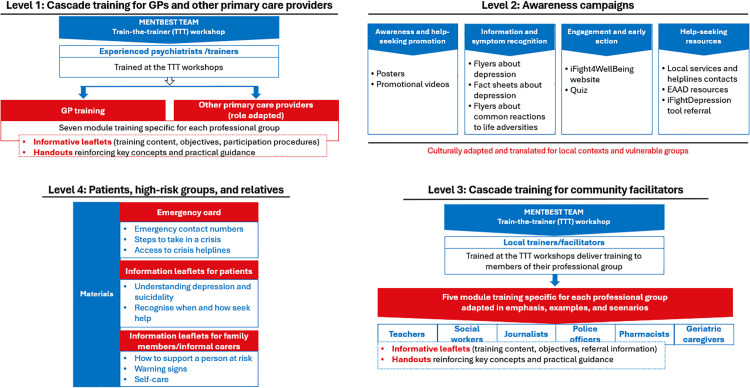
Overview of intervention materials per level.

Materials have been developed for each of the four EAAD intervention levels: 1) Primary health care, 2) General population, 3) Community facilitators, 4) Affected individuals’ and their relatives. To ensure synergistic effects, it is essential that activities across all four levels are implemented simultaneously [57]. Each national team will adapt the training content to cultural and system contexts while maintaining core evidence-based elements of the EAAD model. An overview of the materials can be seen in Fig 3.

Level 1: Training for General Practitioners and Primary Care Providers. As part of the COMBINA intervention, comprehensive training programs were developed for General Practitioners (GPs) and other primary care professionals who treat depression depending on the local context, such as nurses and psychologists. These programs aim to enhance healthcare professionals’ capabilities in diagnosing, managing, and treating depression and suicidal behaviour, particularly among the identified vulnerable populations through appropriate curriculum on knowledge, attitudes and clinical skills. These trainings were crafted to ensure they meet the needs of patients affected by depression and suicidality accessing GPs and other healthcare professionals in the different regions. The trainings last 4–5 hours, with a strong practical component in practising skills learnt. Each country will decide, depending on local structure, whether to accredit the trainings. Trainings will be held by experienced psychiatrists, previously trained by the COMBINA team in specialized Train-the-Trainer (TTT) workshops.

Additional materials include informative leaflets outlining training content, objectives, and participation procedures, and handouts reinforcing key concepts and offering practical guidance during and after the training.

Level 2: Awareness Campaigns. A series of public awareness campaign materials have been co-developed with co-creators to enhance mental health literacy, increase recognition of depressive and subclinical symptoms, reduce stigma, and encourage help-seeking behaviour. These materials have been carefully designed, through engagement with co-creators, to be culturally sensitive, engaging, and easily understandable, drawing on evidence-based communication models for mental health promotion. Non-clinical symptoms were described as “common reactions to life adversities” to normalise these experiences and reduce stigma. This approach aligns with health literacy principles [58], simplifying complex medical language and making it easier for people with varying levels of education to understand and act on early signs of distress.

The materials, which can be seen in more detail in Fig 2, combine several complementary formats, including posters, flyers, fact sheets, and short promotional videos. Together, these materials aim to deliver clear, consistent messages across multiple channels, reaching different segments of the population and aiding both information provision and help-seeking. They are designed as a coherent package, combining concise visual messages, informative written content, and interactive elements. Posters and videos convey core messages in a simple and memorable way, while flyers and fact sheets offer more detailed information and practical advice. Visual identity elements, such a sprout symbol representing “You’re not alone,” are used consistently across formats to increase recognisability and reinforce key messages. The campaign also encourages people, especially young audiences, to share the sprout symbol on social media and messages as a simple act of solidarity and support.

To further promote mental wellbeing and broaden the reach of the intervention, the project has developed a free and openly available internet resource, the “iFight4WellBeing” website. This website has been developed in accordance with the four components of mental health literacy [59,60], providing an accessible, evidence-based online resource that addresses simultaneously subclinical symptoms and broader mental health concerns. The tool complements the community campaign and training activities by offering self-directed guidance and practical exercises in multiple languages. The content is divided into sections: understanding symptoms, a well-being assessment using the Patient Health Questionnaire-4 [61], a 4-week activity planner [62], physical exercise routines [63,64], guidance on sleep regulation [65], relaxation exercises [66,67], and guidance on when to seek professional help.

Level 3: Community Facilitators. Training programs have been developed for community facilitators to enhance awareness, identification, and management of depression and suicidality across different professional sectors. The curriculum builds on the evidence-based EAAD training materials and was adapted through expert consultation and co-creation with national partners to ensure cultural and contextual relevance. The trainings aim to build confidence in recognising signs of distress, initiating supportive conversations, and referring individuals to appropriate services. Content and format were adapted to the specific professional roles and working environments of each group, ensuring relevance and feasibility. Targeted groups included teachers, pharmacists, counsellors, social workers, geriatric caregivers, journalists, police, and clergy, among others. The training lasts for 4–5 hours and is delivered by a mental health specialist, such as a psychiatrist or psychologist.

Level 4: Patients, High-Risk Groups, and Relatives. Psychoeducational materials have been developed to support both patients and their families in managing depression, as can be seen in Fig 2. These include a fact sheet on depression, an information leaflet for family members, and an Emergency Card with contact details for local emergency services and personal emergency contacts.

Furthermore, patients with mild to moderate depression may be referred to a guided online self-management tool for people with mild to moderate depression: the iFightDepression® tool [68,69]. The iFightDepression® tool is part of the EAAD materials and is designed for use by patients with mild to moderate depression, who are referred by trained primary care professionals, and who guide the patient’s use of this self-management tool, based on cognitive behavioural therapy techniques. Referral criteria include: (1) age ≥ 15 years (or younger if deemed appropriate), (2) mild to moderate depression (clinician-assessed), (3) access to internet and email, and (4) informed consent provided. Exclusion criteria are: age < 15 years (unless locally permitted), severe depression, acute suicidality, or current substance abuse.

Cultural Adaptation and Translation. Materials have also been adapted to address the specific needs of each region’s local context and the five vulnerable groups. These adaptations were informed by stakeholder input and co-creation activities to ensure relevance. All materials are translated into the local regional languages: Albanian, Estonian, Greek, Spanish and Catalan. Translations have been validated by native speakers familiar with local mental health contexts, and a translation kit was developed to ensure consistency of key terms, phrases, and concepts across all materials.

All materials have also been translated and culturally adapted for major migrant groups in the participating regions, including the languages Arabic, Chinese, French, Lithuanian, Polish, Russian, Ukrainian, and Urdu. The adaptation process followed a structured framework combining forward translation, expert review, and validation by bilingual mental health professionals to ensure both linguistic accuracy and cultural appropriateness. Key terminology and core messages were standardised across languages to maintain consistency while allowing flexibility for local nuances. For older adults, printed materials have been provided with larger fonts and less text, including paper versions of the iFight4WellBeing resources to ensure accessibility for individuals with limited digital literacy or without digital access. For young people, materials were made visually more engaging with simplified language and age-appropriate vocabulary. Messages have been refined to align with local perceptions and cultural norms.

Regular bi-weekly meetings across countries will take place throughout the intervention period, to collaborate on how the intervention is being delivered in each context and share best practise.

### Implementation process

#### Data collection.

**Fig 4:**
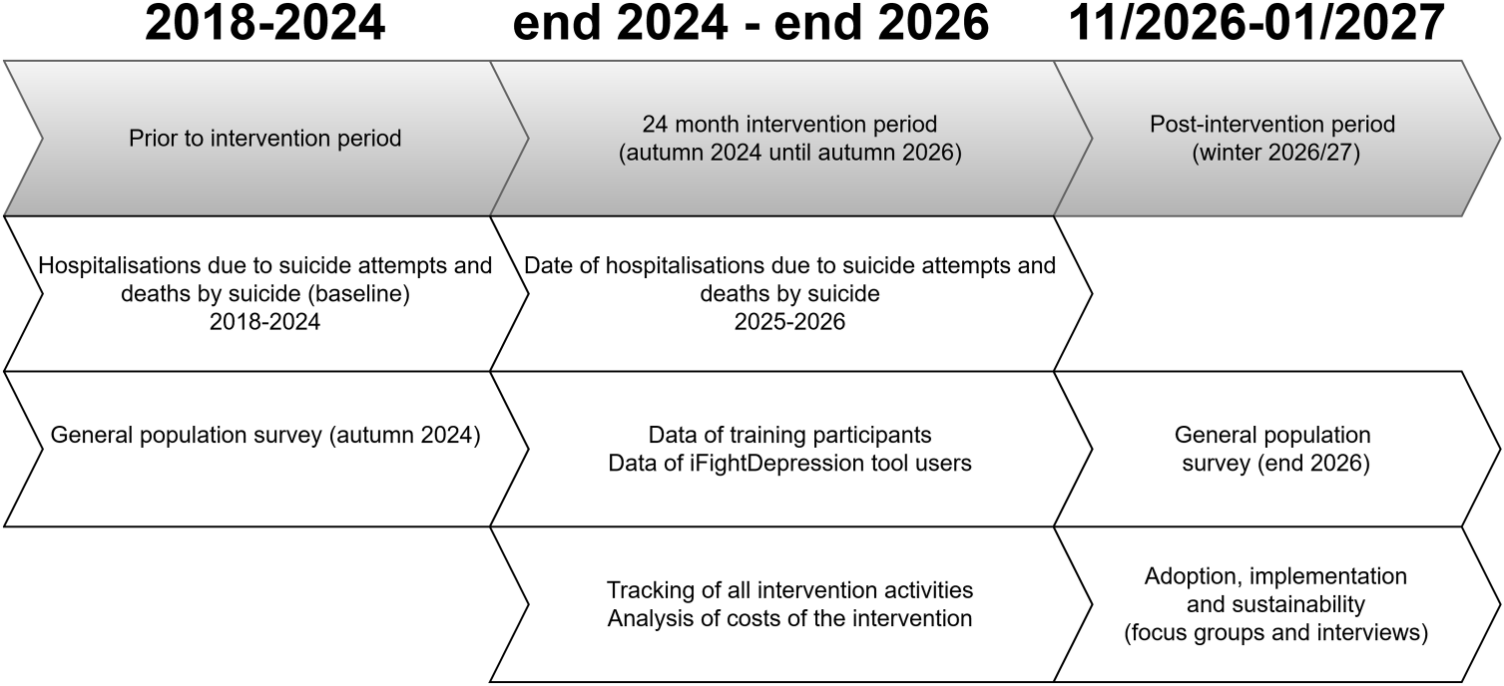
Timeline of the implementation process.

Participant recruitment and data collection for this trial began 24/09/2024 and will be completed by 31/12/2027. Participant recruitment will be completed by 31/01/2027, and data collection will be completed by 31/12/2027. Results will be provided by 31/03/2028. Recruitment began at different timepoints in the countries depending on when ethical approval was received. Recruitment began on 24/09/2024 in Greece, on 01/10/2024 in Estonia, on 07/10/2024 in Albania, on 12/11/2024 in Spain, and on 27/11/2024 in Ireland. The completion point for data collection is the same in all countries: 31/01/2027. Quantitative and qualitative data will be collected to support the outcome, process, and economic evaluations of the COMBINA intervention, as illustrated in Table 3. Please see Fig 4 for an overview of the timeline of the implementation process and different aspects of data collection. To guide the selection of indicators and measures, an Implementation Research Logic Model was developed for the EAAD community-based 4-level intervention and its implementation [75]. This model provides a detailed and comprehensive mapping of: determinants influencing implementation, implementation strategies employed, mechanisms of action, implementation outcomes, and clinical outcomes. An overview of this logic model is available in Supporting Information S2 File.

**Table 3:** Overview of indicators used for the data collection.

Indicator category	Indicator	Data source	Timing of data collection	Where collected
Outcome	Rates of completed suicide	Official statistics from responsible authorities in intervention and control sites	Retrospective and prospective rates from the period 2018–2026	Intervention and control regions
	Rates of hospitalisations due to suicide attempts	Medical records from hospitals in intervention and control sites	Rates from the period 2018–2026	Intervention and control regions
	Knowledge and attitudes related to depression, help-seeking, wellbeing, and symptoms of depression/anxiety	Validated surveys^a-d^ among general population in intervention and control sites	Before and 24 months after implementation	Intervention and control regions
	Competencies of professionals (Level 1 and Level 3 trainings)	Online validated survey^e,f^	Before and immediately after each training session	Intervention regions only (not applicable to control regions)
	Symptoms of depression in iFightDepression tool users	Online validated survey^g^ via iFightDepression tool (no control group)	At registering and 6 weeks later	Intervention regions only (not applicable to control regions)
Process	Reach of the intervention	Monitoring instrument and 4-level intervention tracker completed by research officers	During the 2-year implementation of the intervention	Intervention regions only (not applicable to control regions)
	Adoption, implementation, sustainability of the intervention	Interviews with research officers	After 24 months	Intervention regions only (not applicable to control regions)
	Adoption, implementation, sustainability of the intervention	Focus groups with stakeholders	After 24 months	Intervention regions only (not applicable to control regions)
	Satisfaction and acceptability of professionals (Level 1 and Level 3 trainings)	Online bespoke survey	Immediately after each training session	Intervention regions only (not applicable to control regions)
	Reach of the public campaign	Survey among general population (campaign awareness)	After 24 months	Intervention and control regions
	Reach of the iFightDepression tool	User data from iFightDepression tool and iFight4Wellbeing website	After 24 months	Intervention regions only (not applicable to control regions)
	Acceptability of the iFightDepression tool	Online survey among iFightDepression tool users	6 weeks after registration	Intervention regions only (not applicable to control regions)
	Acceptability of the iFightDepression tool	Online survey among iFightDepression tool guides	After 24 months	Intervention regions only (not applicable to control regions)
	Acceptability of the iFightDepression tool	Focus groups with guides of iFightDepression tool users	After 24 months	Intervention regions only (not applicable to control regions)
Cost	Direct costs of the intervention	Project cost tables, monitoring instrument, and exchanges with research officers	During the 2-year implementation of the intervention	Intervention regions only (not applicable to control regions)
	Costs of health care use and other resources used due to an (attempted) suicide	Estimates from literature, hospital data and expert opinion	Not applicable	Intervention and control regions
	Productivity losses due to an (attempted) suicide	Estimates from the literature, hospital data and expert opinion	Not applicable	Intervention and control regions

Key. ^a^ Depression Stigma Scale (DSS) [70]; ^b^ Attitudes Toward Seeking Professional Psychological Help-Short Form (ATSPPH-SF) [71]; ^c^ World Health Organisation – Five Wellbeing Index (WHO-5) [72]; ^d^ Patient Health Questionnaire 4 (PHQ-4) [61]; ^e^ Revised Depression Attitude Questionnaire (R-DAQ) (Haddad et al., 2015); ^f^ Literacy of Suicide Scale (LOSS) [73]; ^g^ Patient Health Questionnaire-9 (PHQ-9) [74].

### Outcome evaluation

The outcome evaluation will primarily focus on **changes in attempted suicide rates**, operationalised as hospitalisations for intentional self-harm, comparing intervention and control regions over the 2-year implementation period. Monthly hospitalisation data for intentional self-harm will be retrospectively extracted from hospital records covering September 2019 to August 2026, from hospitals serving residents of the intervention and control regions and agreeing to provide data. **Suicides**, including deaths of undetermined intent, will be analysed as a **key secondary outcome**. Annual suicide mortality data will be obtained from national statistical offices for the years 2018–2026 and expressed per 100,000 population by gender.

Other secondary outcomes include changes in public knowledge and attitudes toward depression and its treatment, and mental wellbeing. These will be assessed through general population surveys conducted in both intervention and control regions at baseline and 24-month follow-up, with samples of 500 residents per region. The chosen sample size is consistent with earlier large-scale evaluations of the EAAD multi-level intervention [76], which successfully used regional samples of approximately 500 respondents to detect meaningful changes in depression stigma, attitudes toward help-seeking, and awareness of campaign activities across intervention and control regions. Replicating this sampling approach ensures sufficient power to detect small-to-moderate changes in our outcomes. The samples are stratified by age and gender, following predefined quota for the target population, and recruited through a mix of face-to-face interviews, telephone interviews, and online surveys. Surveys were carried out by external accredited market research agencies, to improve methodological robustness and reduce selection bias. The survey includes sociodemographic and health-related questions, and four validated scales. Below, the English language versions of the scales are described. Validated translations were used for Albanian, Estonian, Greek, and Spanish, and where no validated scale was available, a back translation process was used.

- Wellbeing: World Health Organisation – Five Wellbeing Index (WHO-5) [72]. This is a self-report scale which measures wellbeing through five items scored on a Likert scale from 0 to 6. The raw score is calculated by totalling the numbers of the five answers, and ranges from 0 to 25. To obtain a percentage score ranging from 0 to 100, the raw score is multiplied by 4. A percentage score of 0 represents worst possible level of well-being, whereas a score of 100 represents best possible well-being.
- Depression and anxiety: Patient Health Questionnaire 4 (PHQ-4) [61]. The PHQ-4 is a brief, 4-item self-report instrument that combines the two-item Patient Health Questionnaire depression scale (PHQ-2) and the two-item Generalized Anxiety Disorder scale (GAD-2) into a composite measure of depression and anxiety symptoms. Respondents are asked how often they have been bothered by each symptom over the past two weeks, using a 4-point Likert scale: ‘not at all’ (0), ‘several days’ (1), ‘more than half the days’ (2), and ‘nearly every day’ (3). The total score ranges from 0 to 12, with higher scores indicating greater severity of depression and anxiety symptoms.
- Depression stigma: personal subscale of the Depression Stigma Scale (DSS) [70]. The personal subscale of the Depression Stigma Scale consists of 9 self-report items referencing stigmatised attitudes towards depression. Level of agreement with each statement is scored on a Likert scale of 1–5. A total score is achieved by summing the answers; higher scores indicate greater levels of personal stigma towards depression.
- Attitudes toward seeking help: Attitudes Toward Seeking Professional Psychological Help-Short Form (ATSPPH-SF) [71]. This 10-item self-report scale evaluates attitudes towards help-seeking for mental health difficulties. It is scored on a Likert scale of 0–3, with some answers worded negatively and with inverse scoring. The scores are then summed to provide an overall score, where a higher score indicates a more positive attitude towards help-seeking.

To examine whether the Level 1 and Level 3 trainings reach the desired outcomes, health professionals and community gatekeepers participating in the trainings will be asked to complete the Revised Depression Attitude Questionnaire (R-DAQ) [77] immediately before and after training, and the Literacy of Suicide Scale (LOSS) [73].

Finally, users of the iFightDepression® tool will be asked to complete the Patient Health Questionnaire (PHQ-9) which measures symptoms of depression at registration and six weeks later [74], in order to measure the impact of the tool on depressive symptoms. The PHQ-9 is a nine-item self-report questionnaire evaluating depressive symptoms, scored on a Likert scale of 0–3. Higher scores indicate a greater level of depressive symptoms.

### Process evaluation

The process evaluation will assess key aspects of implementation, such as reach, adoption, implementation, acceptability, and sustainability of the intervention. Multiple data sources will be used to capture these dimensions:

- **Monitoring templates and trackers** to be completed by local research officers to document implementation activities (e.g., alliance functioning, awareness campaigns, training sessions, media engagement).
- **Semi-structured interviews** with local research officers to explore regional differences, facilitators, and barriers.
- **Satisfaction surveys for Level 1 and Level 3 trainees** to assess relevance, usability, and perceived impact of the trainings for professionals.
- **Satisfaction surveys for tool guides, and tool users** to assess relevance, usability, and perceived impact of the iFightDepression® tool.
- **Log data from the iFightDepression® tool** (e.g., number of registrations, repeat visits, total usage time) to evaluate engagement.
- **Focus group with stakeholders** in each country to evaluate the acceptability, implementation process, and sustainability of the intervention.

The design of the data collection instruments is grounded in three widely used implementation science frameworks. The Consolidated Framework for Implementation Research (CFIR) [20,78] guides the identification of barriers and facilitators across five domains (intervention characteristics, inner and outer settings, individual characteristics, and implementation processes) providing a nuanced understanding of contextual influences. The RE-AIM framework [21] informs the evaluation of key dimensions of implementation success, including reach, adoption, fidelity, and maintenance. Finally, the taxonomy of Proctor and colleagues [25] offers a structured set of implementation outcomes, such as acceptability, appropriateness, feasibility, and sustainability, that complement RE-AIM and enable a comprehensive assessment of how the intervention is integrated into practice. Together, these frameworks ensure that the process evaluation captures both contextual determinants and measurable indicators of implementation quality.

### Economic evaluation

The economic evaluation will use a cost-consequence analysis from both healthcare payer and societal perspectives. Costs considered include:

- **Direct implementation costs** estimated using a micro-costing approach [79], which involves detailed tracking of resource use and unit costs.
- **Healthcare costs** related to suicide (attempts), based on published literature and/or hospital data.
- **Other societal or private costs** related to suicide (attempts), based on published literature and expert opinion (e.g., informal care).
- **Productivity losses**, calculated per full day of inpatient stay following a suicide attempt, its follow-up period or productivity loss due to premature mortality in case of a successful attempt.

Where feasible, country-specific cost estimates will be derived using local data sources. Outcomes linked to the cost estimates in incremental cost-effectiveness ratios include hospitalisations due to suicide attempts (primary economic outcome), deaths by suicide, and changes in public knowledge, attitudes, and mental wellbeing, as measured through the general population survey. Reporting will occur conform the CHEERS guidelines [80].

### Ethics

The study was conducted according to the guidelines of the Declaration of Helsinki. The trial will be carried out at all times in strict accordance with the principles of voluntariness, confidentiality and non-harm. Ethical approval was received in each participating country, from the Ethical Committee of the Ministry of Health, Albania (No. 6653, date. 10.09.2024), the Human Research Ethics Committee of the National Institute for Health Development in Estonia (Decision No. 1372, date 19.09.2024), the Ethics in research committee of the Psychology department of the University of York Europe Campus in Thessaloniki (No. 1078, dt 16.07.2024), the Social Research Ethics Committee (SREC) Log No: 2024−227 (date: 29.11.2024; population survey) and Log No: 2025−015 (date: 27/02/2025; trainings) in Ireland, and the Drug Research Ethical Committee (CEIm) of Hospital del Mar in Spain (2024/11534/I; date 06/11/2024). All participants, where data is collected, receive information about the study and provide informed consent before participating. For the evaluations of L1 and L3 training participants, and for patients using the online iFightDepression tool, informed consent was provided by clicking on the box in the electronic form. This format for informed consent was also used for all the general population survey participants in Greece and Albania, and for those responding to the survey online in Estonia, Albania, and Spain. For phone and face-to-face interviews, informed consent was provided verbally in these countries. Verbal consent was witnessed by the evaluator and recorded in the tool, and in Spain voice recordings were also taken of the informed consent. No data was collected from any minors during the course of this study.

### Data management

For this trial, data will be transferred and stored following the principles of the EC Directive on personal data protection and confidentiality, conform the General Data Protection Regulation (GDPR) (EC/2016/679). The general population survey data is anonymous, no personal details are collected, and the data will be collected by external, accredited, market research agencies. For the Level 1 and Level 3 trainings, pseudonymised data will be collected from participants through the LimeSurvey tool (https://www.limesurvey.org/), where the data from responses will be managed by KU Leuven. The patient data for the iFightDepression® tool will be stored securely on a secure server in Germany, and anonymised data will be provided to KU Leuven for analysis.

When processing and analysing the data, measures will be taken to eliminate the risk of identification of individual participants/people in the pseudonymised multi-country dataset, for example by collapsing country of origin categories when there is a small number of individuals in one specific country. Similarly, only aggregated anonymised data will be used in publications referring to the project.

Directive 2001/20/EC (Clinical Trials Directive), Regulation EU No 536/2014 (Clinical Trials Regulation), Regulation EU No 2017/745 (Medical Device Regulation) or Regulation EU No 2017/746 (In-Vitro Diagnostic Medical Devices Regulation) are not applicable to the project. No other additional regulatory documents are relevant for the project.

### Statistical analysis

A range of statistical methods will be employed to analyse the diverse quantitative and qualitative data collected throughout the study. Descriptive statistics (e.g., means, standard deviations, frequencies) will be used across all datasets to summarize key variables. For the survey data, statistical techniques such as Chi-square test, analysis of variance (ANOVA), linear regression, and Pearson correlations will be employed to assess associations between variables and differences across groups (e.g., countries, professional groups).

To assess changes over time and between intervention and control regions, longitudinal and multilevel analyses will be applied. These include a generalised linear mixed model, Binomial and Poisson Regression, Interrupted Time Series Analysis, Linear Mixed Modelling (LMM), and Repeated Measures ANOVA. Missing data in longitudinal models will be handled using the implicit imputation properties of linear mixed models, which use all available data under the missing at random (MAR) assumption and provide unbiased parameter estimates without the need for explicit imputation procedures.

Finally, qualitative data from interviews and focus groups will be audio-recorded, transcribed, and analysed using thematic analysis to identify key patterns and insights.

### Dissemination

The results of this study will be disseminated through scientific publications in high-ranking peer reviewed journals, as well as through scientific conferences, and shared in lay language with stakeholders from the intervention regions. Co-creators will be involved in the dissemination process.

## Discussion

### Regional adaptations

The five COMBINA countries differ in terms of culture, economy, and existing mental health services and infrastructure, creating challenges for the successful implementation of this protocol, and highlighting the need for the protocol to be adaptable to each setting [81]. The project will draw from extensive experience in implementing the Community-Based 4-Level Intervention concept and building local alliances in over 120 regions across 15 countries in order to successfully implement the intervention in the different settings [82].

Mental health services vary significantly across different European countries in terms of care availability and capacity [83]. Implementation of the COMBINA intervention may be affected by variations across the five participating countries in a range of domains. Firstly, in terms of health system organisation and primary care capacity, Ireland and Spain have national suicide prevention strategies and community mental health frameworks that align well with the EAAD model [84,85]. Greece and Albania are currently implementing mental health reforms with variable funding and regional coverage, which may affect institutional buy-in [86,87]. Estonia has robust digital health policies but limited workforce capacity in mental health, posing potential implementation barriers [88,89].

Secondly, there are regional differences in cultural attitudes and stigma. Stigma toward mental illness and help-seeking remains relatively high in Albania and Greece [90,91]. Ireland and Spain demonstrate greater public openness to discussing mental health and support for community-based promotion [92–94]. Social stigma in Estonia, particularly in men, around mental health may be a barrier to help-seeking behaviours [95]. Thirdly, the economic and social context differs across countries. Economic instability and unemployment rates are higher in Greece and Albania, wh.-ich may exacerbate psychological distress but also increase the relevance of preventive initiatives [96,97]. Ireland and Spain face persistent urban–rural inequalities in access to mental health services, while Estonia’s smaller population and high digital connectivity may enable more targeted outreach [98–100].

Furthermore, the 4-level intervention includes several digital components and there are important differences in digital literacy and access across the regions.

Estonia has high digital literacy and extensive integration of online public services, offering fertile ground for digital mental health interventions [98]. Ireland and Spain show moderate to high digital engagement, whereas Greece and Albania have lower digital access and literacy, especially in rural or older populations [101,102], necessitating greater reliance on in-person communication strategies.

Finally, there are differences regarding stakeholder engagement and community infrastructure. Spain and Ireland benefit from well-established community networks and civil society [103,104] organisations that can act as facilitators for local implementation [105]. In contrast, Greece, Albania, and Estonia may rely more heavily on institutional or municipal structures rather than non-governmental organisations, which could influence the reach and flexibility of community engagement activities [106].

Due to these regional differences, this protocol is designed to be flexible, so that the intervention can be adapted to the local context and integrated into the existing services and networks while focusing on the gaps in existing services where the EAAD resources can be of most use, while maintaining the key four-level elements across each country. This cross-country implementation also enables shared learning, allowing adaptation strategies developed in one setting to inform implementation in others.

Operational challenges include variability in availability and quality of data related to deaths by suicide and attempted deaths by suicide, ethical review timelines, and local capacity for training delivery, which were addressed through flexible planning and bi-weekly cross-country coordinating meetings.

### Incorporating subclinical symptoms and syndromes

Widening the EAAD framework to integrate wellbeing promotion and target subclinical symptoms of depression and anxiety may help to engage individuals earlier, before clinical symptoms emerge, and broaden the population impact of the intervention. Community-based approaches, including gatekeeper trainings, the collaboration of local services working together, and connecting people with community resources, have demonstrated effectiveness in improving wellbeing [107]. These aspects are all present in the community-based 4-level intervention and have been adapted to include a focus on wellbeing. Moreover, a key innovation is the development of the iFight4wellbeing website, which offers an engaging, easy-to-use, anonymous interface for mental health promotion [108]. Its design and the fact that no data are gathered, builds user trust, while its evidence-based content ensures relevance and reliability to responsibly meet users’ needs [109]. The tool is incorporated into the gatekeeper trainings and introduced through the COMBINA stakeholder network, facilitating uptake and reducing barriers to implementation [110]. Meanwhile, as digital exclusion can have a negative impact on health [111], attractive flyers were created with the most important information from the iFight4Wellbeing website, including versions in migrant languages and simplified language, for those who are unable to access digital interventions.

### Reaching vulnerable groups

Evidence highlights that certain population groups are particularly vulnerable to poor mental health outcomes, yet they often remain underserved by conventional prevention strategies [112,113]. Therefore, ensuring that interventions effectively reach the five vulnerable groups within the COMBINA project requires strategies tailored to their specific needs and contexts [114]. When such tailoring is implemented, evidence shows that interventions achieve higher levels of engagement, acceptability, and overall effectiveness. For example, for migrant populations, evidence from several meta-analyses shows that culturally adapted mental health prevention programmes achieve better outcomes than non-adapted ones among migrants and ethnic minorities [115]. Similarly, a review found that to engage with hard-to-reach older populations, gaining family support is needed for those of 85 and older, while gaining the support of community leaders is key in engaging with older minority groups [116]. For younger people, co-creation is increasingly being used to ensure relevance and resonance [117]. In the COMBINA project, co-creation is being used to identify how best to reach each vulnerable group, employing strategies such as the cultural adaptation of materials, the use of digital and paper formats, partnering with community figures who already work with these vulnerable groups, and including people with lived experience across different aspects of the intervention.

### Sustainability

Sustainability, the capacity of an intervention to maintain its benefits over time without ongoing external support, is a critical and challenging consideration for community-based mental health initiatives [118,119]. Common barriers include lack of funding, while facilitators include being able to adapt the intervention, collaborating closely with regional health systems, and organisational leadership [120,121]. COMBINA incorporates several features to promote sustainability. Central to this is creating a network with the key stakeholders, including regional health systems and local authorities as well as the community. These networks foster ownership and facilitate long-term integration of the intervention into existing structures. The flexibility with which this intervention is adapted to the local needs of each region also strengthens sustainability. Furthermore, the model includes a “train-the-trainer” approach, where trainers within the community organisations are trained to be able to continue to provide the trainings following the formal implementation period. Similarly, COMBINA is supported by the EAAD framework, which provides a foundation for continued capacity building and knowledge exchange.

### Strengths and Limitations of this Study

Despite these challenges, the COMBINA study has several notable strengths that reinforce its methodological and practical contribution to implementation research. It builds upon the evidence-based EAAD Community-Based 4-Level Intervention and expands its scope to include mental health promotion, addressing both preventive and proactive aspects of mental wellbeing. The multi-level design, spanning primary care, community facilitators, public awareness, and individuals with lived experience, enables a comprehensive assessment of mechanisms operating across population and system levels. Implementation across five culturally and socioeconomically diverse European countries provides a rare opportunity to examine contextual determinants of success, which supports external validity and scalability. The participatory, co-creation approach ensures that intervention materials and activities are contextually relevant, culturally sensitive, and community-owned, promoting sustainability beyond the study period. The use of established implementation frameworks such as RE-AIM, provides conceptual clarity and ensures systematic evaluation of reach, adoption, fidelity, and maintenance. The study’s mixed-methods design integrates outcome, process, and economic evaluations, yielding a holistic understanding of effectiveness and implementation processes. Finally, by targeting vulnerable and underserved populations—such as young people, older adults, migrants, the long-term unemployed, and individuals with lived experience of mental disorders—the project advances equity-oriented mental health research and contributes to reducing disparities across Europe. The COMBINA trial offers valuable insights for policymakers seeking scalable, culturally adaptable mental health interventions. Its multilevel structure and emphasis on vulnerable populations align with WHO and EU priorities for integrated community-based mental health promotion. Moreover, the five participating countries differ considerably in terms of culture, health system organisation, and mental health service capacity, creating substantial contextual variability which increases the generalisability of the findings. However, these differences may influence both implementation fidelity and effectiveness, so to ensure fidelity across sites, a central monitoring framework was developed, incorporating standardized implementation trackers, shared training protocols, and multilingual resource packages.

A key limitation of the study design is the absence of randomisation and blinding, which may introduce selection bias and limit causal inference regarding intervention effects. However, the pragmatic nature of the trial and the use of matched control regions enhance ecological validity and reflect real-world implementation conditions. Although matching and baseline adjustments will be undertaken to control for differences between intervention and control regions, residual confounding cannot be fully excluded. Systematic monitoring of adaptations using the FRAME framework will, however, help interpret these differences. Additionally, ensuring consistent implementation across all intervention sites is challenging due to variation in stakeholder engagement, and local infrastructures, which may affect fidelity and the intensity of delivery. Measurement and data comparability may also be influenced by differences in translation, cultural interpretation, and administration of survey instruments, despite rigorous harmonisation and back-translation procedures. Furthermore, the reliance on self-report data introduces the potential for social desirability bias, particularly in countries where stigma toward mental illness remains high. The study’s medium-term evaluation period may not capture long-term outcomes or sustainability effects, and differences in local funding structures and institutional support may affect reach and data completeness. Given the low frequency of deaths by suicide, it will be challenging to see enough variability in suicide rates to statistically link it to exposures. Finally, because the intervention is community-based and highly visible, spill over effects between intervention and control regions may occur, potentially diluting measurable differences.

## Conclusion

The COMBINA trial aims to build on the existing, evidence-based, community-based 4-level Intervention, to better reach vulnerable groups and include a focus on promoting wellbeing. It constitutes a comprehensive strategy to evaluate the effectiveness, implementation process, and cost-effectiveness of the expanded EAAD intervention across five European countries. Its multi country, prospective design allows for robust comparisons between intervention and control regions, providing evidence on suicide rates, hospitalizations, public attitudes toward depression, and general wellbeing. The combined use of quantitative and qualitative methods ensures a broad understanding, incorporating clinical indicators as well as the experiences of professionals, users, and communities, while the inclusion of an economic evaluation will provide insights into the intervention’s value-for-money and scalability. The implementation protocol includes flexibility to respond to its implementation in five different regional contexts and in vulnerable groups, and sustainability may be supported through networks with local authorities, health systems, and community actors, along with adaptation of the intervention to regional contexts.

## Supporting information

S1 File SPIRIT 2025 checklist of items to address in a randomized trial protocol. (DOCX)

S2 File Specification of all relevant determinants, implementation strategies, mechanisms of action, and expected outcomes related to the expanded 4-level intervention based on the Implementation Research Logic Model of Smith et al. (2020). (DOCX)

S3 File Inclusivity in Global Research form. (DOCX)

## Abbreviations

ANOVA: Analysis of Variance
CFIR: Consolidated Framework for Implementation Research
EAAD: European Alliance Against Depression
EU: European Union
GDPR: General Data Protection Regulation
GP: General Practitioner
ISRCTN: “ISRCTN - the UK’s clinical studies registry”
LMM: Linear Mixed Modelling
RE-AIM: Reach, Effectiveness, Adoption, Implementation, and Maintenance (guidelines for implementation research)
SPIRIT: Standard Protocol Items: Recommendations for Interventional Trials
WHO: World Health Organisation
