# Supplementary material for: COMBINA: The EAAD four-level approach for depression and suicide prevention and wellbeing promotion in the community and vulnerable populations. A cross-country study protocol from the MENTBEST project
Source: PLoS One. 2026 Jul 2;21(7):e0352598. doi: 10.1371/journal.pone.0352598 (PMC13327270; doi:10.1371/journal.pone.0352598)
Supplement: S2 File — (DOCX) [file pone.0352598.s002.docx]

# **Supporting Information 2. Specification of all relevant determinants, implementation strategies, mechanisms of action, and expected outcomes related to the expanded 4-level intervention based on the Implementation Research Logic Model of Smith et al. (2020).**

| **Determinants (facilitators - & barriers +)** | **Intervention strategies** | **Mechanism of action** | **Outcomes** |
| --- | --- | --- | --- |
| **Characteristics of the intervention:**   - Tailored to fit specific local needs (+) - Tailored to the input of relevant stakeholders (+) - Tailored to the input of end-users and service providers (+) - Based on scientific evidence (+)   **Inner setting:**   - Motivation and engagement of municipalities and key services (+/-) - Changes / turnover in service providers (-) - Cooperation with mental health facilities (+/-) - Capacity of mental health facilities (-) - Competition with organisations that pursue similar aims and financial resources (-) - Complementarity with other stakeholder organisations (+/-)   **Outer setting:**   - Aligned with national and local mental health policies and care systems (+/-) - Political shifts in country and/or region (-) - Support received from local government (+) - Interest in society and media for mental health issues (+) - External events overshadowing the intervention (wars, pandemics, natural disasters, economic crises, etc.) (-) - Other competing or complementary online resources used in country/or region (+/-)   **Characteristics of individuals:**   - Motivation of citizens to seek help and stigmatising attitudes concerning mental health - Motivation, interest, attitudes and competencies of professionals   **The implementation process:**   - Involvement, engagement and collaboration of stakeholders, professionals, and volunteers - Use of digital training sessions where needed to reach out to large groups of relevant professionals - One central contact person to streamline communication - Use of digital dissemination tools - Consistent communication and frequent reminders to those involved - Dissemination about the project - Personal / face-to-face contact to increase engagement | **Installation of a regional alliance**   - - Formation of a steering group   - Engagement of existing organizations   - Definition of regional objectives   - Engagement of mayor & spokesperson   - Search for additional funding   - Organization of kick-off event & press conference   **Training of primary care providers, mental health care professionals and community gatekeepers**   - - Training of lecturers   - Development of tailored training materials   - Recruitment of trainees   - Delivery of face-to-face, hybrid or online training   **Raising awareness about depression, subclinical issues & the intervention**   - - Development & distribution of tailored campaign materials   - Establishment of website   - Organization of an opening ceremony & a series of public events   **Strengthening & support of patients & high-risk groups**   - - Online training of professionals to become user guides for the iFD tool   - Referral of persons with needs to iFD tool   - Referral of persons to appropriate care | HC and MHC services and their professionals are brought together in a regional network which may lead to more efficiency and improved care pathways for the treatment of depression. This may result in reduced suicidal behaviour and improved mental wellbeing of citizens in the longer term.  Primary care providers and mental health care professionals who are in close contact with citizens are trained to detect mental health problems and suicidal signals at an early stage and refer them to treatment, resulting in reduced suicidal behaviour and improved mental wellbeing of citizens in the longer term.  Awareness activities are expected to improve the general public’s attitudes towards mental health problems and their treatment. This will make citizens prone to talk more openly about mental health problems and seek help.  Online tools that provide self-help advice to people with subclinical and clinical symptoms of depression will enable more flexible and accessible support to a wider audience in the community and contribute to improved mental wellbeing and reduced symptoms of depression in the longer term. | **Clinical outcomes**   - - Rates of completed suicide   - Rates of suicide attempts   - Knowledge and attitudes towards depression and treatment   - Mental wellbeing   - Symptoms of depression & anxiety   **Service outcomes**   - - Penetration in HC & MHC facilities   - Competencies of trained professionals   **Implementation outcomes**   - - Reach   - Adoption   - Implementation   - Acceptability   - Sustainability   - Costs |

*HC: healthcare; MHC: mental healthcare; iFD tool: iFightDepression tool.*
